# Case report: Splenic artery pseudoaneurysm mimicking a bleeding marginal ulcer in a patient with gastric bypass

**DOI:** 10.1016/j.ijscr.2023.108774

**Published:** 2023-09-02

**Authors:** Agustin Sibona, Keith Scharf

**Affiliations:** Loma Linda University, School of Medicine, 11175 Campus Street, suite 21111, Loma Linda, CA, 92350. USA

**Keywords:** Gastric bypass - gastrointestinal bleeding - splenic artery pseudoaneurysm - bariatric surgery - trans-arterial embolization

## Abstract

**Introduction:**

Upper gastrointestinal (GI) bleeding in patients with roux-en-Y gastric bypass can be difficult to localize. Marginal ulcers are the most common cause, but a broad differential should be maintained in cases of severe bleeding, especially since the stomach and duodenum are not easily accessible by regular upper endoscopy.

**Presentation of case:**

A 38-year-old female with Roux-en-Y gastric bypass presented with abdominal pain and hematochezia. Due to history of smoking and heavy use of ibuprofen, she was initially thought to have a bleeding marginal ulceration. Further investigation with computed tomographic (CT) angiography revealed a splenic artery pseudoaneurysm that had ruptured into a pancreatic pseudocyst, the gastric remnant and the peritoneum. The patient underwent successful treatment with trans-arterial embolization.

**Discussion:**

Splenic artery pseudoanerysms are rare but potentially lethal if unrecognized, particularly in patients with altered foregut anatomy. Their most likely origin is a nearby pancreatic pseudocyst, which erodes into the splenic artery by direct pressure and enzymatic digestion. Bleeding inside the pseudocyst is the most feared complication, resulting in massive intraperitoneal, extraperitoneal or endoluminal hemorrhage. Surgery is particularly challenging due to intense peripancreatic inflammation. *Trans*-Anterial embolization is the preferred treatment modality.

**Conclusion:**

Marginal ulcers continue to be the most common cause of GI bleeding in patients with Roux-en-Y anatomy, although high index of suspicion for alternative diagnosis should be maintained in cases of massive hemorrhage.

## Highlights

• Gastrointestinal bleeding in patients with gastric bypass is a diagnostic challenge.
• Marginal ulcers are the most common cause but rarely hemodynamically significant.
• Splenic artery pseudoaneurysms are rare but potentially lethal.
• *trans*-Arterial embolization is the preferred therapy for stable patients.

## 1 Introduction

Gastrointestinal bleeding in patients with Roux-en-Y gastric bypass can be a diagnostic challenge. Not only a large portion of the foregut structures are not easily accessible by regular endoscopy, but also the most common causes differ from those who have normal anatomy: marginal ulceration is at the top of the differential diagnosis list [1]. Other common causes for late bleeding are gastritis and ulcers in the pouch, gastric remnant or duodenum [2].

Splenic artery pseudoaneurysms are a rare but serious complication of pancreatic pseudocysts. Although most are incidentally detected on computed tomography, their rupture causes massive bleeding inside the gastrointestinal tract or the peritoneal cavity [3]. Mortality has been estimated to approach 90 % if unrecognized [4] and 20 % if treated [5].

We present the case of a female patient who presented to the emergency room with hematochezia and anemia. She reports a remote history of Roux-en-Y gastric bypass, current active smoking and heavy ibuprofen use, so bleeding from a marginal ulcer was suspected. The patient was later discovered to have a splenic artery pseudoaneurysm that ruptured into a pancreatic pseudocyst and her remnant stomach. This case is being reported in line with the SCARE criteria [6].

## 2 Presentation of the case

A 38-year-old female with a remote history of Roux-en-Y gastric bypass presented to the emergency department (ED) with sudden onset of abdominal pain, blood in stool and weakness. Her abdominal pain had started in the morning, accompanied by dizziness, diaphoresis and paleness. She went to the bathroom and passed large amounts of bright red blood per rectum, so she decided to go to the hospital.

**Fig. 1:**
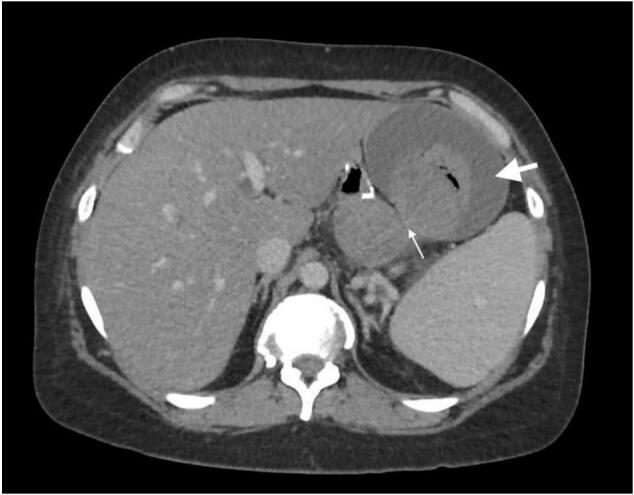
Initial CT scan. There is a fistulous communication between the excluded stomach (small arrow) and blood dissecting the wall components of the remnant stomach (big arrow).

In the ED, vital signs were normal, but hemoglobin was 6.4 mg/dL so 2 units of packed red blood cells were transfused. The patient reported current smoking and heavy use of Ibuprofen for back pain. She also reported a remote history of peptic ulcer disease prior to her bypass. A bleeding marginal ulcer was suspected, so she was admitted with a pantoprazole drip and plans for upper endoscopy the next morning. A computerized tomography (CT) scan of the abdomen and pelvis with contrast was then obtained, reporting “serpiginous hyper dense focus at the jejunonojejunal anastomosis, concerning for hemorrhage” and “likely fistulous communication between the excluded stomach and a 4.6 cm pancreatic pseudocyst, both of which are filled with hyper dense material, which might be hemorrhagic product or necrotic material” (see Fig. 1).

**Fig. 2:**
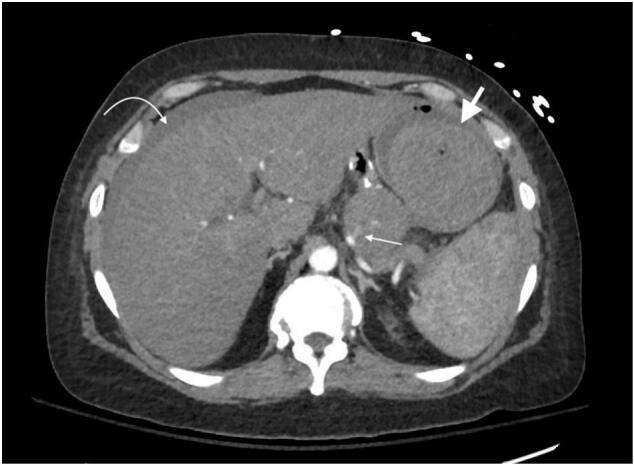
CT angiography. Please observe the active contrast extravasation from the splenic artery (small arrow). There is near complete resolution of the fluid around the gastric remnant (big arrow) and intraperitoneal blood (curved arrow).

On the next morning, the patient became dizzy once again after passing large amounts of bright red blood and clots per rectum. She was hypotensive (blood pressure 70/40 mmHg), tachycardic and diaphoretic. After stabilization with fluids and blood products, CT angiography was performed, reporting “active arterial enhancement within the pancreatic pseudocyst suggestive of active hemorrhage associated with a branch of the splenic artery” and “interval development of hyperdense free intraperitoneal fluid representing hemoperitoneum” (see Fig. 2). After assuring hemodynamic stability, she was taken to Interventional Radiology suite for a splenic angiogram, which demonstrated a pseudoaneurysm arising from the mid splenic artery. The patient underwent successful proximal to mid splenic artery embolization across the pseudoaneurysm's origin. She was discharged home four days after intervention and readmitted on post-procedural day 14 due to left upper quadrant pain and fevers. CT scan showed multifocal scenic infarcts, so she was taken to the Operating Room (OR) for exploratory laparotomy and splenectomy. The patient was discharged home six days after the operation.

## 3 Discussion

The key in this case is to recognize a potential initial misdiagnosis. Although several elements of the history were pointing towards a marginal ulcer as the source of bleeding (gastric bypass, smoking, heavy use of ibuprofen), this pathology rarely causes acute hemorrhagic shock. Since the vital signs were normal during the initial encounter, the focus was placed on her most probable cause of gastrointestinal (GI) bleeding. The incidence of marginal ulcers after bypass has been estimated to be between 0.6 and 25 % [7]. Bleeding per rectum is not a typical presentation but is certainly possible since blood bypasses the remnant stomach and its acidic environment.

Computed tomography showed signs of an alternative diagnosis: although hematic products were spotted at the jejunojejunostomy - which is consistent with bleeding from the marginal ulcer - there was a fistulous connection between the remnant stomach and a pancreatic pseudocyst (see Fig. 1). In addition, there seemed to be a crescent-shaped liquid material dissecting around and in between the wall components of the remnant stomach (see Fig. 1). Upon retrospective review of the images, this was likely blood coming from the splenic artery, draining into the excluded stomach, and also dissecting inside its serosa.

Pancreatic pseudocysts erode into nearby structures by a dual mechanism: direct damage by pancreatic enzymes and local ischemia from direct compression [8,9]. Bleeding into the pseudocyst is one of the most dangerous complications, estimated to happen in 6–10 % of cases [10]. The most common source is the splenic artery, which forms a pseudoaneurysm and then ruptures into the pseudocyst. From here, blood might travel to the gastrointestinal tract, peritoneum or retroperitoneum [11].

This patient had a second episode of profound hypotension, which was perceived as a “double rupture” sign. Although this phenomenon responds to a completely different etiology and pathophysiology (true splenic artery aneurysmal bleeding from the lesser sac into the peritoneum), it served the purpose of reorienting attention to alternative diagnoses. We hypothesize the “second rupture” happened after blood, which had initially dissected inside the stomach wall, ruptured into the peritoneum. This is evidenced by the subsequent presence of intraperitoneal blood and almost complete resolution of the fluid around the gastric remnant (see Fig. 2).

The preferred treatment for a stable patient is angiographic embolization [3]. If done in a controlled setting, the success rate of this procedure ranges between 90 % to 96 % [10,12,13]. Surgery is reserved for hemodynamically unstable patients or failure of trans arterial embolization (TAE). The surgical approach involves ligation of the splenic artery proximal and distal to the bleeding; however, this can be challenging as there is typically intense peripancreatic inflammation. Distal pancreatectomy and/or splenectomy might also be indicated. For patients with gastric bypass, this would also involve mobilization of the roux limb in order to access the gastric remnant and retroperitoneum. This maneuver could lead to roux limb ischemia with subsequent need to major bariatric surgical revisions.

The rate of complications from TEA is estimated to be between 3 and 18 % and includes pancreatitis, pancreatic necrosis, splenic infarction and bowel ischemia [14,15]. The patient in this case developed a splenic infarction with intractable pain and fever. She required an exploratory laparotomy with splenectomy. Although she eventually required an open splenectomy, embolization was still effective at addressing her bleeding and achieving stabilization.

## 4 Conclusion

Gastrointestinal bleeding in patients with Roux-en-Y gastric bypass represents a diagnostic and therapeutic challenge. Endoscopy is limited by difficult access to most of the foregut structures. Although marginal ulcers continue to be the most common cause, a high index of suspicion for and alternative diagnosis is required in cases of massive bleeding. CT angiography is of great value since it can localize the bleeding source while facilitating subsequent endovascular therapeutic strategies.

## Consent

Written informed consent was obtained from the patient for publication of this case report and accompanying images. A copy of the written consent is available for review by the Editor-in-Chief of this journal on request.

## Declaration of competing interests

The authors declare that they have no known competing financial interests or personal relationships that could have appeared to influence the work reported in this paper.
